# The Best Material from the VII Congress of Russian Biophysicists

**DOI:** 10.3390/ijms25074016

**Published:** 2024-04-04

**Authors:** Anastasia A. Anashkina, Stepan S. Dzhimak

**Affiliations:** 1 Laboratory of DNA-Protein Interactions, Engelhardt Institute of Molecular Biology, Russian Academy of Sciences, 119991 Moscow, Russia; anastasia.a.anashkina@mail.ru; 2 Laboratory of Problems of Stable Isotope Spreading in Living Systems, Federal Research Center the Southern Scientific Center of the Russian Academy of Sciences, 344006 Rostov-on-Don, Russia; 3 Physics and Technology Faculty, Kuban State University, 350040 Krasnodar, Russia

The purpose of this Special Issue is to demonstrate the current state of research in the field of biophysics in the Russian Federation. It includes articles that describe some of the results presented at the VII Congress of Russian Biophysicists.

The work of the congress was divided into plenary, sectional and poster presentations. The congress program included sections on molecular biophysics, including the structure and dynamics of biopolymers and biomacromolecular systems, cell biophysics, membrane and transport processes, the mechanisms of energy transformation, bioenergy, molecular motors, biomechanics, biological mobility, the biophysics of complex multicomponent systems, math modeling, bioinformatics, biophotonics, photobiology, photosynthesis, bioluminescence, photoreception, optogenetics, the mechanisms of action of the physicochemical factors in biological systems, environmental biophysics, medical biophysics, neurobiophysics, biophysical education and new methods in biophysics. Throughout the congress, particular attention was paid to the physical mechanisms at work in the biological processes occurring at various levels of the organization of living matter.

The influence of physicochemical factors on living systems is traditionally considered at this congress, with special attention being paid to the study of their mechanisms of action. In Lobyshev V.I.’s report, arguments have been made in favor of the fact that water and the aquatic environment are sensors of weak interactions [1].

A number of reports were devoted to studies on the influence of stable isotopes on living systems. In particular, Yaglova N.V. noted that the processes involved in the formation of lymphocytes in the thymus are highly sensitive to short-term decreases in the deuterium content of the body. A decrease in deuterium stimulates the proliferation of lymphoblasts in the thymus and slows down the migration processes of differentiated T cells. The resulting increase in the formation of T-cytotoxic lymphocytes demonstrates the role of deuterium in the selection of double-positive thymocytes [2].

In addition, it was found that both an increase and decrease in the deuterium content in the body of laboratory animals led to the same changes in the functioning of the pituitary–thyroid axis. The nature of these identified changes indicates the sensitivity of the thyroid gland to modifications of the isotopic composition of fluids in the body [3]. Other authors have noted that the long-term consumption of water depleted in deuterium before hypoxic exposure (exposure to an amnesic factor) has a pronounced protective, anti-amnestic effect [4]. It has been established that, in a deuterium-depleted environment, the membrane potential of the mitochondria of cerebellar neurons decreases [5]. In Koltover V.K.’s report, studies were presented on the catalytic effects of magnetic isotopes of magnesium (^25^Mg) and zinc (^67^Zn) on the enzymatic hydrolysis of ATP by myosin. The rates of ATP hydrolysis in environments containing the magnetic isotopes ^25^Mg and ^67^Zn turned out to be 40–50% higher than the rates in environments with non-magnetic isotopes of these elements [6].

Another widely discussed issue at the congress was the mechanisms of the influence of magnetic fields on living systems. In his report, Krylov V.V. described in detail the dependence of circadian biological rhythms on slow fluctuations of the earth’s magnetic field [7,8]. A number of reports have presented arguments in favor of the idea that the main mechanism of action of low-frequency magnetic fields on living systems is associated with the production of reactive oxygen species [9,10].

A number of reports presented the results of studies on the influence of metal nanoparticles on biological objects, from the level of DNA to mammals [11,12,13,14].

In the field of biomechanics, Kuchumov A.G. presented solutions to the current problems in cardiovascular surgery using computational fluid dynamics methods [15,16]. This work is an excellent example of collaboration between specialists in the field of mathematical modeling, numerical methods and medicine to solve the pressing problems in biophysics.

The largest number of reports at the congress were on the topic of medical biophysics. These reports covered a wide range of topics, from the development of methods for studying oxidative stress in neurodegenerative diseases [17], and neuroprotective drugs [18], to research in the field of protecting the body from the development of diabetes by blocking the formation of excess amounts of the protein VDAC1 using specific blocking molecules and genetic engineering methods [19]. In addition, in her report, Brazhe N.A. presented the finding that astrocytes, rather than neurons, may be a suitable “target” both for the prevention of age-related changes in the body and for the treatment of various types of neurodegeneration [20].

Thus, all areas of biophysical research were widely covered in the reports presented at the congress. A special mention should be made of contemporary achievements in the field of understanding the physical mechanisms and processes involved in biological systems, as well as the enormous progress seen in the development of biophysical experimental methods [21].

The next congress of Russian biophysicists will be held in 2027 in St. Petersburg.

## References

[1] Lobyshev V.I. (2023). Water as a Sensor of Weak Impacts on Biological Systems. Biophys. Rev..

[2] Yaglova N.V., Obernikhin S.S., Timokhina E.P., Nazimova S.V., Yaglov V.V. (2022). Reactive Alterations in Thymic Lymphocytopoiesis to Short-Term Decrease in Deuterium Content in the Body. Bull. Exp. Biol. Med..

[3] Yaglova N.V., Obernikhin S.S., Timokhina E.P., Yaglov V.V. (2021). Response of Pituitary—Thyroid Axis to a Short-Term Shift in Deuterium Content in the Body. Bull. Exp. Biol. Med..

[4] Kozin S.V., Kravtsov A.A., Zlischeva E.I., Shurygina L.V., Malyshko V.V., Moiseev A.V., Elkina A.A., Baryshev M.G. (2020). The Influence of a Deuterium Depleted Drinking Diet on the Functional State of the Central Nervous System of Animals in Hypoxia. Biophysics.

[5] Kozin S.V., Kravtsov A.A., Elkina A.A., Zlishcheva E.I., Barysheva E.V., Shurygina L.V., Moiseev A.V., Baryshev M.G. (2019). Isotope Exchange of Deuterium for Protium in Rat Brain Tissues Changes Brain Tolerance to Hypoxia. Biophysics.

[6] Koltover V.K. (2023). Magnetic Isotope Effects and Nuclear Spin Catalysis in Living Cells and Biomolecular Motors: Recent Advances and Future Outlooks. Biophys. Rev..

[7] Krylov V.V., Sizova A.A., Sizov D.A. (2022). Effects of Hypoxia and Hypomagnetic Field on Morphometric and Life-History Traits in Freshwater Cladoceran Daphnia Magna. Water.

[8] Krylov V.V., Izvekov E.I., Pavlova V.V., Pankova N.A., Osipova E.A. (2022). Magnetic Fluctuations Entrain the Circadian Rhythm of Locomotor Activity in Zebrafish: Can Cryptochrome Be Involved?. Biology.

[9] Tekutskaya E.E., Gusaruk L.R., Ilchenko G.P. (2022). The Effect of an Alternating Magnetic Field on the Chemiluminescence of Human Peripheral Blood Mononuclear Cells and the Production of Pro-Inflammatory Cytokines. Biophysics.

[10] Shaev I.A., Novikov V.V., Yablokova E.V., Fesenko E.E. (2022). A Brief Review of the Current State of Research on the Biological Effects of Weak Magnetic Fields. Biophysics.

[11] Kasyanenko N.A., Baryshev A.V., Bakulev V.M. (2021). Conformational Changes of High-Molecular-Weight DNA upon Binding to Noble Metal Nanoparticles in Solution. Russ. Chem. Bull..

[12] Malyshko V.V., Basov A.A., Dorohova A.A., Moiseev A.V., Dyakov O.V., Pavlyuchenko I.I., Storozhuk A.P. (2023). Dynamics of Indicators of the Prooxidant-Antioxidant System In the Blood and Exudate Under the Treatment Of Purulent Wounds With Negative Pressure and Silver Nanoparticles. Med. News N. Cauc..

[13] Kopytov G.F., Malyshko V.V., Basov A.A., Moiseev A.V., Vlasov R.V., Frolov V.Y., Shashkov D.I. (2022). Cyclic Freezing Effect on Silver Nanoparticle Adsorption on Polished Collagen Fiber. Russ. Phys. J..

[14] Kuznetsov M., Kolobov A. (2023). Mathematical Modelling for Spatial Optimization of Irradiation during Proton Radiotherapy with Nanosensitizers. Russ. J. Numer. Anal. Math. Model..

[15] Kuchumov A.G., Doroshenko O.V., Golub M.V., Saychenko N.D., Rakisheva I.O., Shekhmametyev R.M. (2023). Numerical Method for Geometrical Feature Extraction and Identification of Patient-Specific Aorta Models in Pediatric Congenital Heart Disease. Mathematics.

[16] Kuchumov A.G., Khairulin A., Shmurak M., Porodikov A., Merzlyakov A. (2022). The Effects of the Mechanical Properties of Vascular Grafts and an Anisotropic Hyperelastic Aortic Model on Local Hemodynamics during Modified Blalock–Taussig Shunt Operation, Assessed Using FSI Simulation. Materials.

[17] Kalinichenko A.L., Jappy D., Solius G.M., Maltsev D.I., Bogdanova Y.A., Mukhametshina L.F., Sokolov R.A., Moshchenko A.A., Shaydurov V.A., Rozov A.V. (2023). Chemogenetic Emulation of Intraneuronal Oxidative Stress Affects Synaptic Plasticity. Redox Biol..

[18] Kozin S., Kravtsov A., Ivashchenko L., Dotsenko V., Dzhimak S., Aksenov N., Vashurin A., Ivlev V., Baryshev M., Bespalov A. (2023). Structure and Neuroprotector Properties of a Complex Compound of Lithium with Comenic Acid. Int. J. Mol. Sci..

[19] Belosludtsev K.N., Serov D.A., Ilzorkina A.I., Starinets V.S., Dubinin M.V., Talanov E.Y., Karagyaur M.N., Primak A.L., Belosludtseva N.V. (2023). Pharmacological and Genetic Suppression of VDAC1 Alleviates the Development of Mitochondrial Dysfunction in Endothelial and Fibroblast Cell Cultures upon Hyperglycemic Conditions. Antioxidants.

[20] Popov A., Brazhe N., Morozova K., Yashin K., Bychkov M., Nosova O., Sutyagina O., Brazhe A., Parshina E., Li L. (2023). Mitochondrial Malfunction and Atrophy of Astrocytes in the Aged Human Cerebral Cortex. Nat. Commun..

[21] Penkov N.V. (2023). Terahertz Spectroscopy as a Method for Investigation of Hydration Shells of Biomolecules. Biophys. Rev..

